# Interlocus gene conversion explains at least 2.7 % of single nucleotide variants in human segmental duplications

**DOI:** 10.1186/s12864-015-1681-3

**Published:** 2015-06-16

**Authors:** Beth L. Dumont

**Affiliations:** Initiative in Biological Complexity, North Carolina State University, 112 Derieux Place, 3510 Thomas Hall, Campus Box 7614, Raleigh, NC 27695-7614 USA

**Keywords:** Gene conversion, Polymorphism, Global pairwise alignment, 1000 Genomes, Recombination, Segmental duplication, Gene duplication

## Abstract

**Background:**

Interlocus gene conversion (IGC) is a recombination-based mechanism that results in the unidirectional transfer of short stretches of sequence between paralogous loci. Although IGC is a well-established mechanism of human disease, the extent to which this mutagenic process has shaped overall patterns of segregating variation in multi-copy regions of the human genome remains unknown. One expected manifestation of IGC in population genomic data is the presence of one-to-one paralogous SNPs that segregate identical alleles.

**Results:**

Here, I use SNP genotype calls from the low-coverage phase 3 release of the 1000 Genomes Project to identify 15,790 parallel, shared SNPs in duplicated regions of the human genome. My approach for identifying these sites accounts for the potential redundancy of short read mapping in multi-copy genomic regions, thereby effectively eliminating false positive SNP calls arising from paralogous sequence variation. I demonstrate that independent mutation events to identical nucleotides at paralogous sites are not a significant source of shared polymorphisms in the human genome, consistent with the interpretation that these sites are the outcome of historical IGC events. These putative signals of IGC are enriched in genomic contexts previously associated with non-allelic homologous recombination, including clear signals in gene families that form tandem intra-chromosomal clusters.

**Conclusions:**

Taken together, my analyses implicate IGC, not point mutation, as the mechanism generating at least 2.7 % of single nucleotide variants in duplicated regions of the human genome.

**Electronic supplementary material:**

The online version of this article (doi:10.1186/s12864-015-1681-3) contains supplementary material, which is available to authorized users.

## Background

**Fig. 1:**
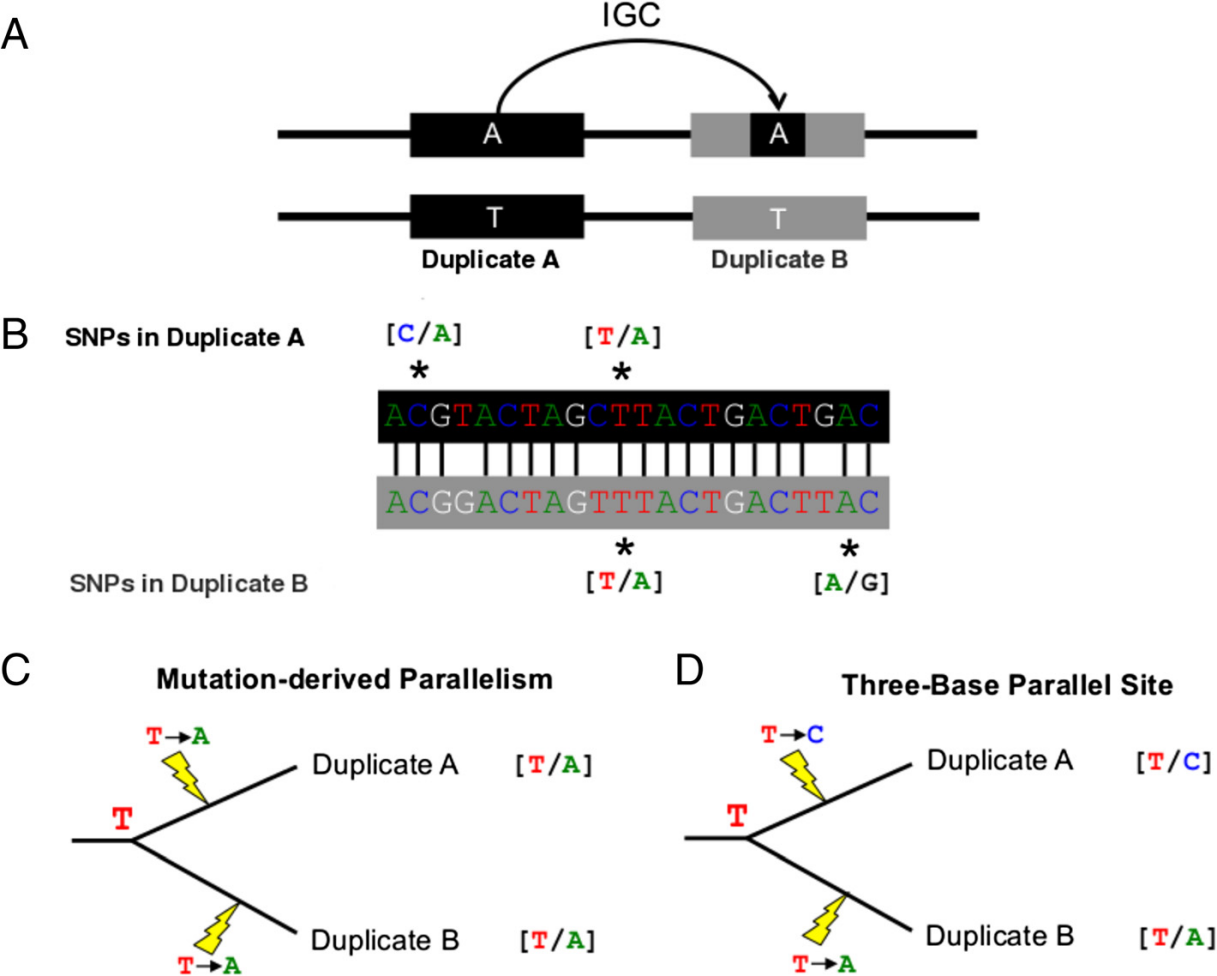
Detecting signals of historical interlocus gene conversion in polymorphism data. **a** An IGC event can transfer an allele from one paralog to another, resulting in shared polymorphic sites or “parallelisms”. **b** To systematically identify such sites, high-quality paralog alignments can be integrated with dense polymorphism data to identify aligned positions that are polymorphic in both paralogs and segregate identical alleles. **c** Such sites may also arise by parallel mutation events that occurred independently during the independent evolution of each paralog. However, parallel mutation should more frequently result in aligned polymorphic positions that segregate alternative alleles (**d**)

Segmental duplications (SDs) are among the most rapidly evolving and dynamic loci in the human genome [1, 2]. These loci are operationally defined as sequences greater than 1 kb in length with over 90 % sequence similarity to a locus elsewhere in the genome [3]. Ectopic recombination between homologous SDs can give rise to large deletions, duplications, inversions, and translocations, including structural mutations associated with human disease [4–6] and rearrangements that may have contributed to the evolution of uniquely human traits [7–10]. One subtle, yet ubiquitous, outcome of non-allelic homologous recombination is interlocus gene conversion (IGC), or the unidirectional transfer of sequence from one SD to a paralogous SD (Fig. 1a). In this manner, IGC functions as a “copy-and-paste” mechanism that imparts two characteristic signatures on the evolution of duplicated sequences. First, IGC decreases divergence between paralogous SDs. The active exchange of variants between duplicates can drive their concerted evolution [11–13] and may even permit the retention of functional similarity between ancient SDs [14]. Second, IGC increases haplotype diversity within duplicated sequences [15, 16], thereby expediting their adaptive evolution and promoting the maintenance of exceptionally high levels of allelic diversity [15–18]. On the other hand, IGC can also introduce deleterious alleles into functionally important genomic contexts. Indeed, IGC is a well-established mechanism of human disease [19, 20], accounting for approximately 1 % of *de novo* disease alleles [21].

Although a handful of exceptional gene families show signals consistent with frequent IGC [22–24], overall, IGC appears to have had a modest effect on patterns of between paralog divergence in the human genome. Benevoy and Drouin (2009) identified longer-than-expected stretches of perfect sequence identity in pairwise paralog alignments, a genetic signature indicative of historical IGC. The authors concluded that 0.88 % of duplicated coding positions in the human genome have likely been converted by IGC [25]. McGrath *et al.* (2009) used a gene-tree species-tree reconciliation strategy to estimate that 2.16 % of duplicated sites in the human genome have been directly converted [26]. Jackson *et al.* (2005) and Dumont and Eichler (2013) used a third approach based on the identification of fine-scale switches in the phylogenetic tree relating paralogs to derive estimated conversion rates of ~4-5 % [27, 28]. Together, these diverse methods converge on a set of common conclusions: the rate of IGC inferred from patterns of paralog divergence is too low to mar the true evolutionary history of most duplicated loci in the human genome, and estimates of divergence times based on sequence comparisons between duplicated loci are usually not deflated by IGC-driven sequence homogenization.

Naively, the limited footprint of IGC on patterns of between paralog divergence in the human genome may also be predicted to extend to its effects on within paralog polymorphism. However, there are at least two reasons to speculate that IGC may have left a more pronounced stamp on levels of within paralog diversity in the human genome. First, the human population has experienced rapid, super-exponential population growth over the last ~100 generations [29]. As a result, most segregating variants in human populations are young, low frequency alleles that are unlikely to have fixed between paralogs. By definition, these segregating variants cannot be used to characterize the impact of IGC on patterns of between paralog divergence, although they may contain information about how this mechanism affects levels of within paralog diversity. Second, many IGC-derived alleles are potentially deleterious [19–21]. Although low fitness genotypes may contribute to transient polymorphism, negative selection should effectively purge deleterious alleles before they leave a footprint in patterns of sequence divergence.

One powerful approach for identifying IGC signals embedded in DNA diversity data is to mine population genetic datasets for one-to-one paralogous positions that are polymorphic for identical alleles [24, 30–33] (Fig. 1b). Approximately 35 % of segregating sites in the first five exons of the human *RHCE* gene are also polymorphic at corresponding positions in the paralogous *RHD* locus [24], and over 10 % of variants in both the pregnancy-specific glycoprotein gene cluster [28] and the human luteinizing hormone/chorionic gonadotropin β gene cluster [34] are shared among paralogs. These case studies highlight the important contributions of IGC to patterns of segregating diversity within single gene families, but it remains unclear how IGC’s effect on diversity at these extraordinary loci extrapolates to its role as a general mechanism of DNA variation across duplicated regions of the genome. Despite the availability of whole-genome sequences from large population samples, the genome-wide mutagenic impact of IGC on human polymorphism has never been directly quantified.

Toward this aim, I leverage well-annotated segmental duplications in the human genome and SNP calls from population genomic sequences generated by the 1000 Genomes Consortium to systematically catalog 15,790 pairs of shared polymorphic positions at one-to-one paralogous sites. I show that these paralogous SNP pairs are most parsimoniously interpreted as the outcome of historical IGC events, rather than the consequence of parallel mutation events. Together, my analyses demonstrate that a small, albeit significant, fraction of variants in duplicated sectors of the human genome have arisen by the recombinogenic process of IGC rather than by conventional point mutation.

## Results

IGC between duplicated loci can introduce shared polymorphisms at one-to-one paralogous positions (Fig. 1). Using SNP calls from 1,058 low coverage whole genome sequences released by the 1000 Genomes Project and 48,931 global pairwise alignments between well-annotated paralogous sequences in the human reference genome, I identified 48,996 duplicated single nucleotide positions segregating identical alleles. Over 7,000 of these parallel SNP pairs overlap regions where the alignment between paralogs is potentially low quality (n = 7,026; see Methods). Excluding these possible alignment artifacts leaves 41,970 putative parallel polymorphic SNP pairs in the human genome (Table 1), including 2,683 sets of ≥3 paralogous SNPs that segregate identical alleles. Together, SNPs at these positions (n = 68,568) account for 9.18 % of all 1000 Genomes SNPs intersecting regions of high quality alignment between duplicated sequences (n = 747,278 total SNPs; Table 1; Additional file 1: Table S1).

**Table 1:** Number and frequency of parallelisms in human segmental duplications

QC filter	Number of parallelisms	Number of higher order parallelisms	Number of SNPs in parallelisms	Number of SNPs in SDs	Percentage of SD SNPs in parallelisms
No filters	48,996	3,227	78,532	1,216,383	6.46
High quality alignment	41,970	2,683	68,568	747,278	9.18
Uniquely mapping SNPs + High quality alignment	30,729	1,982	50,413	610,166	8.26
Non-CpG Sites + Uniquely mapping SNPs + High quality alignment	15,790	606	28,747	554,600	5.18

For ease, I will refer to a pair of parallel polymorphic SNPs as a *parallelism*. I use the term “parallel SNP” to refer to either of the two constituent SNPs in a parallelism.

### Eliminating likely false positive parallelisms due to multiple mappings

Short sequence reads derived from duplicated regions may map to multiple loci in the genome and lead to false positive genotype calls. In particular, redundant or incorrect mappings between highly identical duplicates could generate artificial parallelisms and lead to the spurious inference of IGC. To mitigate the effects of this important and likely source of error, I eliminated SNP calls associated with reads that map to multiple locations in the human reference genome (see Methods). This filtered call set contains 610,166 SNPs in duplicated regions that are uniquely taggable in the context of short-read paired-end mapping. Within this set of uniquely mapping SNPs, there are 30,729 parallelisms composed of 50,413 parallel SNPs that together account for 8.26 % of all uniquely mapping SNPs within SDs (Table 1).

Not surprisingly, the majority of parallelisms that are eliminated by the unique read mapping filters occur in high-identity duplicated regions that cannot be reliably distinguished by short reads. Specifically, 55.9 % of redundantly mapping parallelisms lie in duplicated regions with >95 % sequence identity, and 10.7 % fall in regions with >99 % pairwise sequence identity (PSI). In contrast, only 42.8 % and 3.6 % of uniquely mapping parallelisms are located in paralogous sequences with >95 % and >99 % PSI, respectively (Additional file 2: Figure S1).

To confirm that uniquely mapping short paired-end reads can accurately identify SNPs in duplicated regions, I validated a subset of parallelisms using 15× whole genome sequence and ~538,000 fosmid clone insert sequences from a Gujarati Indian individual (GM20847) not included among the 1000 Genomes samples [35]. Because of their large insert sizes (~38 kb), fosmid clone sequences will map to a single position in the genome with near certain probability, thereby eliminating the multiple placement problem associated with short read shotgun sequencing. A total of 1,579 parallelisms were identified from SNP calls based on the whole genome sequence of GM20847, including 117 that were also identified in the 1000 Genomes SNP data. The majority of parallelisms identified in GM20847 (n = 1,459; 92.4 %) contain variants that are not found in the 1000 Genomes dataset. Although a subset may involve SNPs that are specific to the Gujarati Indian population, many are likely artifacts arising from incorrectly mapped short reads, cryptic structural variation, or genotyping errors introduced by modest sequence coverage.

 Of the 117 parallelisms shared between the GM20847 and 1000 Genomes samples, eleven are covered by at least five fosmid clones at both parallel positions (Additional file 3: Table S2). At these loci, the probability that only one allele is represented among the sequenced clones is at most 2 × 0.5^5^ = 0.0625, assuming no allele bias. These clone-based sequences validate ten of the eleven shared parallelisms (90.9 %; Additional file 3: Table S2), providing a compelling proof-of-principle demonstration for the power of uniquely mapping short reads to identify SNPs in a paralog-specific context. The sole exception involves the putative parallelism between SNPs rs115842861 and rs2736944, for which only one allele is represented among the five fosmid clones overlapping each SNP.

### Controlling for the effects of parallel mutation

An alternative biological interpretation for shared polymorphisms is independent mutation events to identical nucleotides at paralogous sites (Fig. 1c). Multiple, independently derived estimates of the *de novo* rate of IGC in the human genome converge on a per site, per generation frequency of ~10^−6^ [28, 36–38]. This rate is approximately 100-fold higher than the *de novo* point mutation rate [39–43], and even exceeds the rate of mutation at hypermutable CpG dinucleotides by an order of magnitude [39, 42, 44, 45]. Although IGC is the most parsimonious interpretation for shared polymorphisms, I conservatively exclude 14,939 parallelisms containing SNPs within hypermutable CpG dinucleotides from downstream analyses. The final, filtered dataset includes 15,790 parallelisms composed of 28,747 unique polymorphic sites (Table 1).

**Fig. 2:**
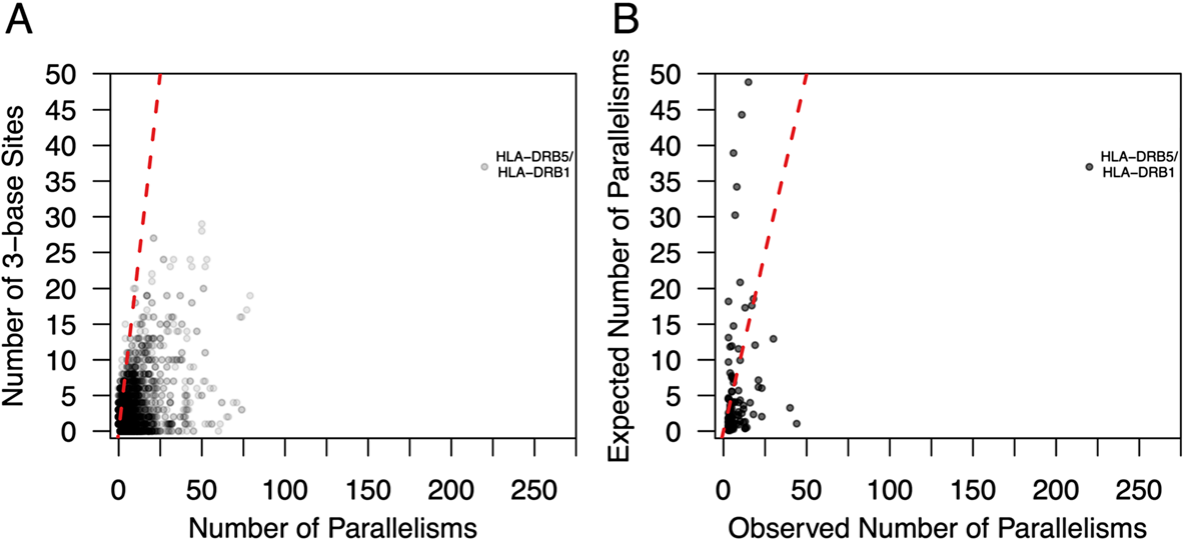
The number of parallelisms exceeds expectations under mutation-only null models. **a** The observed number of parallelisms in each pairwise paralog alignment is plotted against the number of one-to-one aligned polymorphic sites that segregate alternative alleles. **b** The observed number of parallelisms in a random set of 100 paralog alignments is plotted against the expected number of parallelisms derived from coalescent simulations. In both plots, the dashed red line corresponds to the null expectation assuming equal mutation rates to all nucleotides (y = 2×)

To further assess the extent to which mutation may confound the signal of IGC, I conducted two additional analyses on this final parallelism dataset. First, assuming that a given nucleotide mutates to any other nucleotide with equal probability, two out of every three parallel mutation events should yield aligned, paralogous SNPs that segregate alternative alleles (Fig. 1d). In contrast to this prediction, pairwise SD alignments harbor a clear excess of shared polymorphic sites (15,790 non-CpG parallelisms versus 6448 non-CpG paralogous sites with 3 bases). Over 94 % of paralog alignments contain more parallelisms than expected given the observed number of one-to-one SNP pairs with three nucleotides (Fig. 2a). Second, I performed a series of coalescent simulations [46] to determine the expected frequency of parallelisms in a subset of 100 randomly selected SD alignments (Additional file 4: Table S3; see Methods). For 66 of the 100 SD alignments considered in this simulation study, significantly more parallelisms were observed than expected if mutation were the sole evolutionary force creating them (Fig. 2b; Additional file 4: Table S3).

### Properties of parallelisms are consistent with the mechanism of IGC

**Fig. 3:**
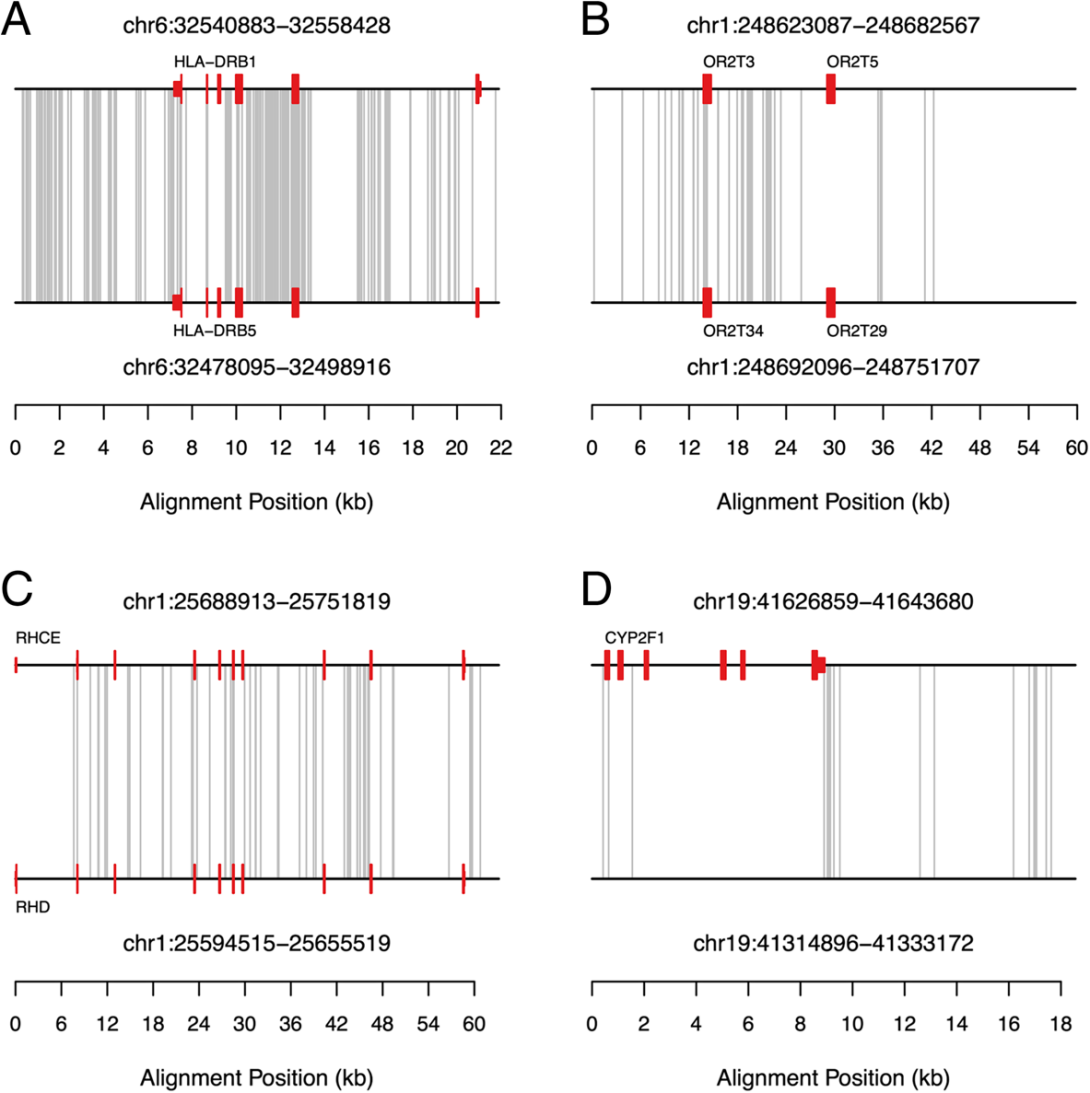
The distribution of parallelisms across four pairwise alignments. Aligned sequences are depicted as horizontal black lines. Protein coding features are represented by thick red boxes, with untranslated sequences marked by the thinner rectangles. The positions of parallelisms within each alignment are shown by vertical gray lines connecting the two aligned sequences. **a** Alignment between duplicons spanning *HLA-DRB1* and *HLA-DRB5* on 6p21. **b** Alignment of segmental duplications that include olfactory gene clusters in the subtelomere of the short arm of chromosome 1. Several clusters of parallelisms between these duplicons are evident, including six parallelisms between *OR2T3* and *OR2T34* and a group of four in the ~5 kb region downstream of *OR2T29* and *OR2T5*. **c** Alignment between the tandem inverted *RCHE*/*RHD* duplication on 1p36. **d** Alignment involving a duplicon spanning the 3’ end of *CYP2F1*. A cluster of parallelisms in the middle of this alignment includes sites in the 3’ UTR of this gene

The distribution of parallelisms in the human genome recapitulates several patterns that are consistent with IGC, providing additional evidence for their origin via this mechanism. First, IGC is more frequent between intra-chromosomal duplicates than between inter-chromosomal SDs [20, 25, 27, 47]. Whereas 38.5 % of analyzed SD alignments involve sequences located on the same chromosome, 67 % of parallelisms are between intra-chromosomal SNPs (n = 10,587). This represents a significant excess over the number expected if parallelisms occur randomly within duplicated sequence space (*P* < 0.001 in comparison to 1000 datasets composed of randomly designated “parallelisms”). Second, many parallelisms concentrate within previously characterized IGC hotspots in the human genome, including the Rhesus blood group antigens *RHD* and *RHCE* on 1p36.11 [24, 48, 49], the pregnancy-specific glycoprotein cluster on chromosome 19q13 [28], olfactory receptors [22], and the *HLA* locus [23, 50, 51] (Additional file 1: Table S1; Fig. 3). Third, parallelisms strongly cluster within many paralog alignments (Additional file 5: Table S4; Fig. 3), a pattern reflecting the possible co-transmission of multiple linked sites within a single IGC track or the cumulative effects of multiple overlapping tracks from independent IGC events initiated at a common hotspot locus. Finally, 89.3 % of parallel SNP pairs segregate in the same 1000 Genomes population, consistent with the intra-genomic transfer of one allele to a second locus via IGC. This percentage exceeds that in 1000 simulated datasets composed of allele frequency matched SNP pairs drawn at random from the full 1000 Genomes SNP call set (Range: 82.7 %-84.0 %; *P* < 0.001).

### Hotspots of interlocus gene conversion

I have uncovered evidence for historical IGC involving 5.2 % of uniquely mapping, non-CpG SNPs in SDs. Accounting for nested redundancies in the data due to sets of >2 paralogous SNPs (see Methods), I estimate that 2.7 % of SNPs in duplicated regions of the human genome have arisen via the mutagenic action of IGC (Table 1). This overall percentage conceals considerable variation among individual paralog pairs in the human genome (Additional file 6: Table S5). In fact, the majority of paralog alignments harbor no high-confidence parallelisms (84.7 %), whereas IGC between other duplicated sequences is rampant, accounting for upward of 10 % of all uniquely mapping variants (Additional file 6: Table S5). For example, there are three high-confidence parallelisms (six parallel SNPs) between the tandem duplications spanning exons 4–7 of the *DUOX1* and *DUOX2* genes on 15q21.1. Over 20 % of all SNPs observed in regions of high quality alignment between these duplicons are involved in a parallelism, suggesting that ~10 % of variants in these genes have arisen from historical IGC events (Additional file 6: Table S5). Similarly, of the 87 SNPs observed in high quality aligned regions between *IFITM2* and *IFITM3* on 11p15.5, 18 are implicated in parallelisms (Additional file 6: Table S5). Approximately 10 % of SNPs in these duplicated genes are also likely the direct by-product of IGC ((18 ÷ 2)/87 = 0.1034).

In addition to these gene-level IGC hotspots, there are 606 SNPs (2.1 % of all uniquely mapping parallel SNPs) that have potentially seeded new SNPs at multiple paralogous acceptor loci (Additional file 7: Table S6). Many of these higher order parallelisms involve sites in tandem duplicate clusters, a structural confirmation associated with frequent, recurrent non-allelic homologous recombination [26, 27, 52–54]. In particular, 33.4 % of higher order parallelisms involve ≥3 SNPs within 5 Mb of each other (Additional file 7: Table S6). The pregnancy-specific glycoprotein (PSG) gene cluster on 19q13.2 provides an especially striking example. *PSG* genes are abundantly expressed in the placenta during pregnancy and are vital for safeguarding the developing fetus from infectious agents in the maternal bloodstream [55, 56]. Across the 11 tandemly duplicated genes in this family, there are 24 higher-order parallelisms, including three 4-dimensional parallelisms and one parallelism involving five SNPs (Additional file 8: Figure S2). Many of these higher dimensional parallelisms involve coding SNPs, raising the possibility that IGC between *PSG* paralogs has played a role in the adaptive protein evolution of this gene family.

### Population-specific signals of historical IGC

Many parallelisms are composed of at least one SNP that is private to one of the 14 populations represented in the 1000 Genomes SNP data (n = 2920; 18.5 %; Additional file 9: Table S7). More than half of these “private parallelisms” are specific to one of the three surveyed African populations (55.4 %), an expected consequence of higher DNA diversity in these populations. At the extremes, the Luhya population of Kenya has 865 private parallelisms, whereas the Iberian population of Spain has just 17 parallel polymorphic sites that are not observed in any other population. These population-specific parallelisms likely reflect historical IGC events that occurred in the population harboring the private allele. Private alleles are often evolutionarily young [57], a point that supports the interpretation that most population-specific parallelisms have probably arisen from IGC events in recent human history.

Several genes harbor multiple population-specific parallelisms (Additional file 9: Table S7). Of the 195 high confidence parallelisms identified in *DPP6*, eight are unique to the Yoruba, seven to the Luhya, and four are exclusive to the British population. This gene shows signals of recent adaptive protein evolution [58] and variants in the regulatory region of *DPP6* are associated with ventricular fibrillation [59]. There are two CEPH-private parallelisms in *SRR*, a gene that has been previously implicated in schizophrenia [60]. Such patterns point to locus and population-specific effects of IGC on genetic diversity, and suggest potential differences in susceptibility to IGC-mediated disease in individuals with alternative ancestries.

## Discussion

I have identified a set of SNP pairs in the human genome that represent outcomes of historical IGC events. Although my approach cannot polarize these SNPs into donor and acceptor sites, I can confidently deduce that one of the two constituent parallel SNPs arose as a consequence of the mutagenic action of IGC, not point mutation. As more high-depth whole genome sequences for diverse unrelated individuals come offline [61], methods based on sequence read depth at uniquely mappable positions in the human genome can be used to systematically distinguish between donor and acceptor alleles (*e.g.* [47]).

Despite this limitation, the sites I identify collectively comprise a catalog that will empower future investigations on IGC. This resource will be especially useful for deriving biologically relevant parameter values and fine-tuning evolutionary models to accurately reflect observed patterns of human polymorphism in duplicated genomic regions (e.g., [62]). In addition, empirical analyses on the spatial distribution of parallelisms across the genome and their relationship with respect to various sequence properties may provide insights into the molecular mechanism of IGC, including whether the process is biased toward transmission of G and C alleles, like allelic gene conversion. Although none of the parallel SNPs in high quality parallelisms are known causal disease variants, newly discovered disease variants in SDs can be rapidly crosschecked against this database to deduce the molecular processes responsible for their origin.

This analysis has focused exclusively on SNPs, but the approach taken here can be readily extended to include other variant types such as indels and multinucleotide variants. Moreover, this general method for identifying IGC signals can be applied to other species with population genomic data. This straightforward extension will facilitate comparative studies on the evolution of the mechanism of IGC, including comparisons of its impact on DNA sequence variation in diverse organisms.

Although differences in power between divergence-based and polymorphism-based methods for detecting IGC signals make it difficult to directly compare estimates [30], results from this analysis suggest that human polymorphism data is not a richer reservoir of historical IGC signals. The estimated 2.7 % of SNPs in human SDs that have arisen from IGC is commensurate with estimates derived from fixed differences between paralogs in the human genome (Range: 1-5 % of duplicated positions) [25–28]. Three considerations, however, suggest that this polymorphism-based figure is an underestimate of the true number of IGC-derived sites in SDs. First, although some parallelisms may be false positives due to the confounding effects of mutation, my quality control pipeline for filtering SNPs likely discarded many more false negative signals. In particular, restricting my focus to only non-CpG parallelisms eliminates 48.6 % of uniquely mapping parallelisms with high quality alignments (Table 1). SNPs at CpG dinucleotides account for 44 % of all 1000 Genomes SNPs in SDs, indicating that this class of highly mutable sites is not markedly enriched in the context of parallelisms. Second, owing to ambiguities in read placement, highly identical duplicated sequences cannot be queried using short read sequencing data, even though existing evidence points to the possibility that these sequences experience the highest *de novo* rates of IGC [20, 47, 63]. As a result, the current analysis has focused disproportionately on the detection of exchange events between more divergent SDs that have potentially experienced lower rates of historical IGC.

Finally, this study has systematically mined the human genome for one specific signature associated with IGC: shared, parallel SNPs. IGC events that span fixed differences between paralogs can also lead to *de novo* SNPs. If IGC occurred recently, the minor SNP allele will likely correspond to the fixed base at a paralogous position. Across the pairwise alignments analyzed here, there are 70,619 SNPs with minor allele frequency ≤0.01 at which the minor allele is fixed at a paralogous position. This corresponds to 5.8 % of all SNPs in duplicated sequence space and 9.0 % of all SNPs in segmental duplications (SDs) with minor allele frequency ≤0.01 (70,619 of 783,168 SNPs in SDs with MAF ≤ 0.01). However, alternative evolutionary scenarios can generate an identical genetic signal, without a requirement for IGC. For example, positive directional selection or random drift could lead to the near fixation of a new allele in one duplicate. Complex population genetic simulations are needed to determine the relative fraction of these signals that is attributable to IGC, an effort that will ultimately yield a more accurate estimate of IGC’s impact on diversity in the human genome.

Regardless of the precise number of segregating variants due to IGC, the finding that a non-negligible fraction of SNPs within human SDs are not due to mutation *per se*, but rather the mutagenic action of IGC bears directly on the continued study of these functionally significant regions of genome. First, as a consequence of variant input via IGC, SDs may harbor more diversity than expected under neutral evolutionary models. Elevated diversity relative to null expectations is commonly a signature of loci evolving under positive Darwinian selection [64–66] and is also a hallmark of balancing selection [67, 68]. Thus, it is imperative that efforts to infer selection in duplicated sequences either (i) explicitly account for the confounding role of IGC or (ii) demonstrate a lack of evidence for IGC. Second, empirical population genetic parameter estimates for these regions, such as the population mutation rate (θ) and the number of segregating sites (S), will be inflated by mutational input from IGC. Third, the fate of deleterious and disease-associated alleles in SDs may not be appropriately modeled using standard predictive frameworks. A variant that is deleterious in the context of one paralog may persist in populations at IGC-selection balance if it is neutral (or beneficial) in the context of a second paralog [19, 21]. Such a scenario is especially likely to hold for parallelisms that involve one genic variant and one variant in a non-coding pseudogene. Additionally, the mechanism of IGC may constitute a form of meiotic drive that can override the effects of purifying selection on deleterious alleles, enabling them to persist in populations at higher-than-expected allele frequencies or even fix [69]. Finally, as a consequence of IGC, an allele that physically maps to a genomic locus in one individual’s genome may map to a distinct position in the genome of other individuals. This positional ambiguity poses a major obstacle to *de novo* assembly and accurate short-read mapping in genomic regions with recurrent IGC. The continued development of novel experimental methods with the precision to query variants within high copy genomic sequences will not only meet this challenge [35, 70], but will also foster deeper understanding of the mutational impact of IGC in the human genome.

## Conclusions

Here, I have used publicly available population genomic data from the 1000 Genomes Project in conjunction with well-annotated duplicated sequences to identify 15,790 shared, polymorphic SNPs between one-to-one aligned duplicated positions in the human reference genome. I have carefully shown that these parallelisms are not the consequence of redundant read mappings in multi-copy genomic regions and that they cannot be explained by parallel mutation events. I conservatively estimate that at least 2.7 % of SNPs in duplicated regions of the human genome have arisen as the consequence of IGC, not point mutation. These findings underscore the importance of an often over-looked mechanism of human genomic diversity – including possible disease alleles – and bear on the interpretation of polymorphism patterns across the ~5 % of the human genome that lies in segmental duplications.

## Methods

### Sequences and alignments

Sequence coordinates for paralogous sequence pairs in the human reference genome (hg19) were obtained from the genomicSuperDups table downloaded from the UCSC Table Browser [71]. The corresponding nucleotide sequences were extracted from the reference assembly and aligned using the *stretcher* program implemented in the EMBOSS suite (version 6.3.1; [72]). This dataset consists of 48,931 global pairwise alignments between segmental duplications mapped to whole-chromosome scaffolds in the human reference assembly (average alignment length (±1 standard deviation): 13,490 bp (±28,300); average PSI: 0.94(±0.027)). Although there is considerable redundancy among these alignments owing to the nested nature of human SDs, aligned sequences cumulatively cover 163 Mb of sequence (5.5 % of the human reference genome).

Regions of low quality or uncertain alignment were identified using several complementary methods. First, I computed average PSI in 100 bp non-overlapping sliding windows across each alignment, and masked windows with PSI <2 standard deviations below the alignment-wide average PSI. Second, I used the method of Han *et al.* [73] to identify shorter, poorly aligned windows across these alignments. Briefly, for each site in an alignment, I extracted the four flanking sites (two sites on each side) to obtain a 5-site sub-alignment centered on the focal position. Sub-alignments with Hamming distance >2 between the two sequences (including indels) were masked. Finally, I excluded sites within 10 bp of an indel and masked the first and last 100 sites in each alignment to eliminate potentially poorly aligned regions at the alignment edges. These conservative quality control steps eliminated an average of 2845 sites per alignment (on average, 26.9 % of the total alignment length) and reduced the number of surveyed bases from 159.5 to 129.6 Mb.

### Identification of Parallelisms and Uniquely Mapping SNPs

Masked alignments were integrated with genotype calls from low-coverage short-read whole-genome sequencing of 1,058 unrelated individuals carried out by the 1000 Genomes Project Consortium (Phase 3; [74]) to identify parallel polymorphic sites. Briefly, for each pair of aligned sites in a given alignment, I determined whether both positions correspond to biallelic SNPs ascertained in the 1000 Genomes SNP call set and, if so, whether both SNPs segregate identical alleles, accounting for the strand orientation of paralogous sequences in the alignment. I focus exclusively on SNPs and exclude indels and complex variants from this analysis.

To identify the subset of Phase 3 1000 Genomes SNP calls that are derived from reads that map to single positions in the hg19 reference genome, I first extracted all sequence reads overlapping SNPs in SDs from the bam alignment files. I then eliminated any SNP calls associated with reads mapping to (i) multiple best hit loci (*i.e.*, the X0 field is >1) or (ii) >5 suboptimal hit positions (*i.e.*, the X1 field is >5). I further required that the alignment between a read and any suboptimal hit contain at least two mismatches more than the number of mismatches in the alignment of the read to its optimal placement. These filtering steps leverage information from mate pairs, such that if one read in the pair maps redundantly and the other maps uniquely, the paired read unit is considered to map to a single locus in the reference genome. Note also that these quality control steps are performed on a per-sample basis. Reads overlapping a given SNP may map redundantly within the genome of one sample, but map uniquely in other sequenced individuals. In these instances, only SNP calls from the latter individuals are retained for downstream analysis.

### Clone sequence analysis

Fosmid clone pool sequences from a Gujarati Indian individual (GM20847) were downloaded from the NCBI short read archive as unaligned sam files (project accession SRP004325 [35]), converted to pool-specific fastq files, and mapped to the hg19 human reference genome using *bwa* [75]. SNP calls from conventional whole-genome shotgun sequencing of this genome were obtained from the author’s website (http://krishna.gs.washington.edu/indianGenome/).

The standard samtools/bcftools pipeline (v1.1.19a; [76]) was used to call bases in each clone pool at positions corresponding to SNPs identified in the diploid genome sequence from this individual. Only reads with mapping quality score >15 and that mapped in a proper pair were used for allele calling.

### Coalescent simulations

Coalescent simulations were performed on a subset of 100 paralog alignments using Hudson’s *ms* [46]. Simulations were informed by empirical summary statistics estimated from the 1000 Genomes data and model major events in human demographic history. First, for both paralog sequences in a given alignment, I conditioned simulations on the observed number of segregating sites in the sequence (*i.e.* the number of SNPs across the locus observed in the 1000 Genomes dataset). For example, if the sequence harbors 300 segregating sites, I simulated coalescent datasets with 300 variable sites. Second, I assume a single population with a static population size for most of its history (N_e_ = 10,000), and model a strong recent population expansion that initiated 100 generations ago (coefficient of exponential population growth α = 920; [29]). I did not attempt to account for more complex aspects of population structure, migration, or intralocus recombination, although previous studies suggest that doing so may yield simulated datasets that better capture nuanced patterns of human DNA diversity [77, 78]. For both sequences in a paralog alignment, I generated 1000 simulated datasets, each composed of 2116 haplotypes (*i.e.,* twice the number of sequenced individuals in the 1000 genomes data). Specifically, the executed commands are:

- ./ms 2116 1000 -s *S1* -eG 0.0025 0 -G 920
- ./ms 2116 1000 -s *S2* -eG 0.0025 0 -G 920

where *S1* and *S2* are the observed number of segregating sites in the two paralogous sequences. For each simulation replicate, I determined the number of variable sites present at the same position in both paralogs. Assuming a Jukes-Cantor model of nucleotide substitution, one-third of these “two-hit” sites will segregate the same alleles and manifest in sequence data as mutation-derived parallelisms.

### Estimating the fraction of SNPs due to IGC

At each parallelism, I assume that one SNP arose by *de novo* mutation and that an IGC event subsequently transferred the new to the paralogous position. However, owing to the presence of higher order parallelisms in the human genome, the number of SNPs in human SDs due to IGC is not simply equivalent to the number of parallelisms. For an *n*-dimensional parallelism, (*n*-1) SNPs have presumably arisen via IGC following a single mutation event at one paralog. Therefore, the number of SNPs within duplicated regions of the genome that are due to IGC is given by: ${\displaystyle \sum_{n=2}^N}k\left(n-1\right)$, where *k* is the observed number of *n*-dimensional parallelisms.

### Statistical analyses

All statistical analyses were implemented in the R Environment for statistical computing [79].

To test whether observed parallelisms are enriched in specific genomic contexts, I generated 1000 null datasets by randomly sampling 15,790 (*i.e.*, the total number of high-confidence parallelisms identified after stringent filtering) unique one-to-one aligned positions from the set of 48,931 paralog alignments. Sampling was agnostic to the variant status of the underlying sites.

I used an *ad hoc* clustering test to evaluate the null hypothesis that parallelisms are uniformly distributed across alignments. To ensure adequate statistical power, this analysis was restricted to the subset of large paralog alignments (>10 kb) with >10 parallelisms (n = 386). The observed number of parallelisms in 1 kb non-overlapping window across each paralog alignment was compared to the uniform expectation (number of parallelisms/number of 1 kb windows) using a Chi Square goodness-of-fit test. *P*-values were calculated from the empirical Chi Square distribution (degrees of freedom = number of non-overlapping windows in alignment – 1).

## Availability of supporting data

The data sets supporting the results of this article are included within the article and its additional files.

## Additional files

Additional file 1: Table S1. High confidence, uniquely mapping parallelisms in the human genome. Additional file 2: Figure S1. Pairwise sequence identity and the cumulative frequency of parallelisms composed of uniquely and non-uniquely mapping SNPs. The cumulative frequencies of parallelisms that pass (dashed blue line) and fail (solid red line) the filtering criteria for uniquely mapping SNPs (see main text) are plotted as a function of pairwise sequence identity between duplicates. As expected, there is an excess of parallelisms involving SNPs that cannot be uniquely mapped in duplicated genomic compartments with high sequence similarity. Additional file 3: Table S2. Validation of 1000 Genomes parallelisms using a fosmid clone pool sequencing resource derived from a Gujarati Indian Individual (GM20847). Additional file 4: Table S3. Expected number of parallelisms derived from coalescent simulations. Additional file 5: Table S4. P-values from a test of the null hypothesis that parallelisms are uniformly distributed across alignments. Additional file 6: Table S5. Fraction of SNPs in parallelims for a given paralog pair. Additional file 7: Table S6. Higher order parallelisms. Additional file 8: Figure S2. Higher-order parallelisms across the tandem PSG duplication cluster. This cluster contains 11 genes, including several that are processed as alternate transcripts. Faint blue lines connect the positions of complex parallelisms involving polymorphic sites in three PSG paralogs. Three 5th and one 6th-order parallelism are shown with bold dark blue, green, orange, and red lines, respectively. Additional file 9: Table S7. Population-specific parallelisms.

## Abbreviations

SD: Segmental duplication
IGC: Interlocus gene conversion
SNP: Single nucleotide polymorphism
PSI: Pairwise sequence identity
