# Supplementary figures and images for: Interlocus gene conversion explains at least 2.7 % of single nucleotide variants in human segmental duplications
Source: BMC Genomics. 2015 Jun 16;16(1):456. doi: 10.1186/s12864-015-1681-3 (PMC4467073; doi:10.1186/s12864-015-1681-3)

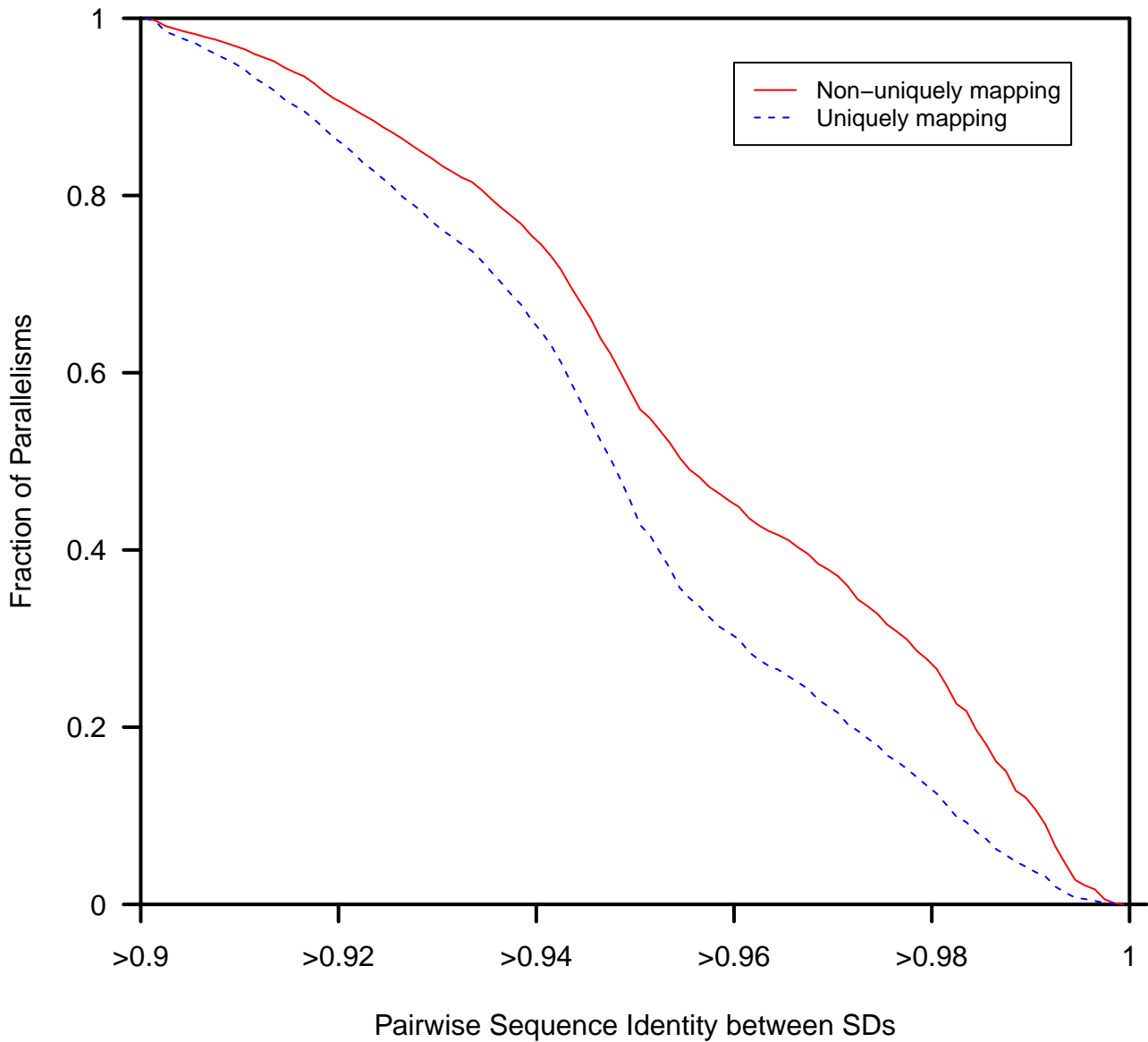

Supplement: Additional file 2: Figure S1. — Pairwise sequence identity and the cumulative frequency of parallelisms composed of uniquely and non-uniquely mapping SNPs. The cumulative frequencies of parallelisms that pass (dashed blue line) and fail (solid red line) the filtering criteria for uniquely mapping SNPs (see main text) are plotted as a function of pairwise sequence identity between duplicates. As expected, there is an excess of parallelisms involving SNPs that cannot be uniquely mapped in duplicated genomic compartments with high sequence similarity. [file 12864_2015_1681_MOESM2_ESM.pdf]

chr19

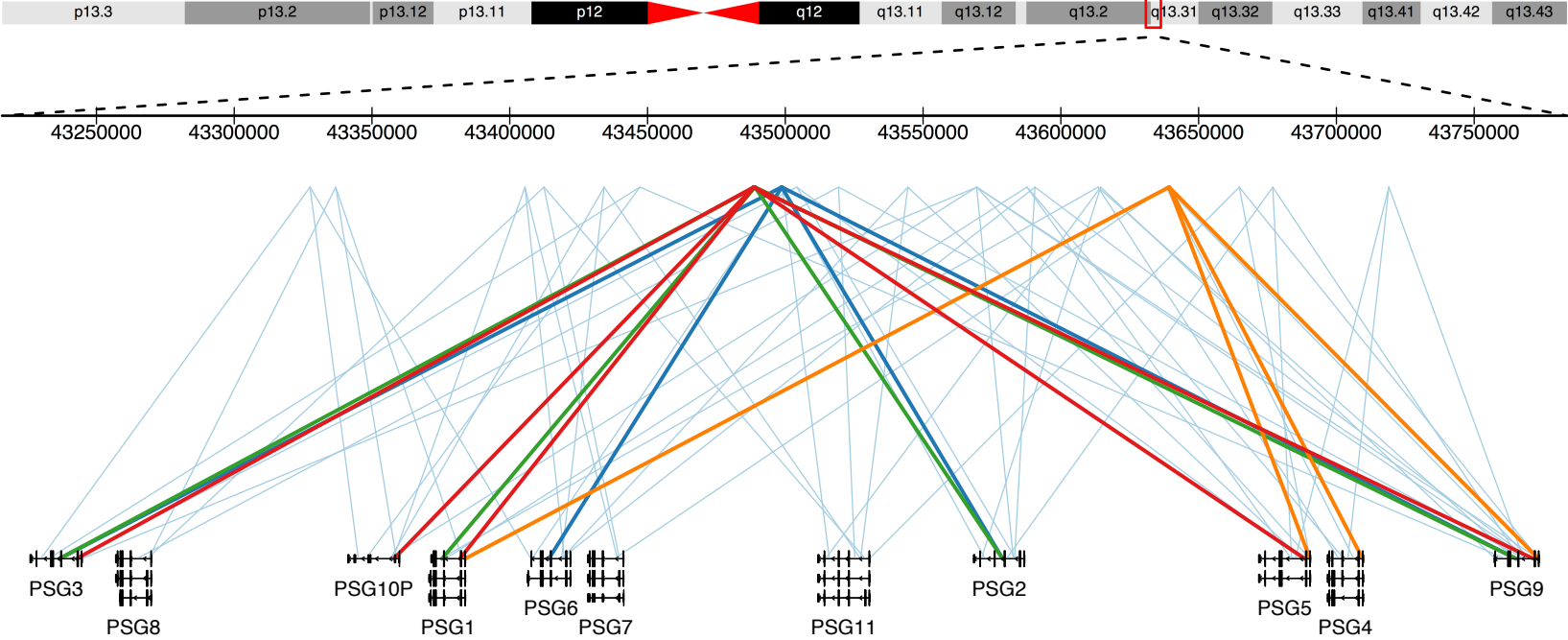

Supplement: Additional file 8: Figure S2. — Higher-order parallelisms across the tandem PSG duplication cluster. This cluster contains 11 genes, including several that are processed as alternate transcripts. Faint blue lines connect the positions of complex parallelisms involving polymorphic sites in three PSG paralogs. Three 5th and one 6th-order parallelism are shown with bold dark blue, green, orange, and red lines, respectively. [file 12864_2015_1681_MOESM8_ESM.pdf]
